# Neurobrucellosis: A Case Report from Himachal Pradesh, India, and Review of the Literature

**DOI:** 10.1155/2016/2019535

**Published:** 2016-10-13

**Authors:** Sujeet Raina, Ashish Sharma, Rajesh Sharma, Amit Bhardwaj

**Affiliations:** ^1^Department of Medicine, Dr. RPGMC, Tanda, Kangra 176001, India; ^2^Department of Neurology, Dr. RPGMC, Tanda, Kangra 176001, India

## Abstract

Human brucellosis is a multisystem disease that commonly presents as a febrile illness along with variable spectrum of clinical manifestations. Neurological complications include encephalitis, meningoencephalitis, radiculitis, myelitis, peripheral and cranial neuropathies, subarachnoid hemorrhage, and psychiatric manifestations. We report a case diagnosed as neurobrucellosis who presented with fever and bilateral upper motor neuron symptoms and signs along with bilateral sensorineural deafness. Diagnosis was confirmed by Rose Bengal Test (RBT) and standard tube agglutination test (SAT).

## 1. Introduction

Brucellosis is the commonest bacterial zoonosis and causes more than 500 000 human infections per year worldwide [1]. The disease has a widespread geographic distribution and is labelled as regionally emerging zoonotic disease [2]. It also comes under the WHO list of neglected tropical zoonotic infection. Brucellosis has a variable clinical manifestation due to extensive involvement of organ systems during infection. Neurobrucellosis is a complication of systemic brucellosis infection. The frequency of neurobrucellosis has been reported as 5–7% in the literature [3]. Neurological complications include encephalitis, meningoencephalitis, radiculitis, myelitis, peripheral and cranial neuropathies, subarachnoid hemorrhage, psychiatric manifestations, brain abscess, and demyelinating syndrome [3, 4]. Human brucellosis has been reported from different states of India. The seroprevalence of brucellosis among occupationally exposed human beings was observed to be 6.66% in Himachal Pradesh, India [5]. Rural population is predominantly agrarian society linked with animal husbandry and shepherding in the state. Migratory pastoralism is very common in the Himalaya and a number of nomadic communities practise this migratory system of goat and sheep rearing in Himachal Pradesh, India. Despite well documented seroprevalence, human brucellosis is less commonly reported from the state [6]. Disease is usually not considered as a cause of meningitis in this region which leads to missed or delayed diagnosis. This could be because of the lack of awareness, suspicion, and diagnostic facilties at the health provider's end. We report a case of neurobrucellosis who presented with fever and bilateral upper motor neuron symptoms and signs along with bilateral sensorineural deafness. Diagnosis was based on consistent clinical features, radiological imaging, positive serum Rose Bengal Test (RBT) and serum standard tube agglutination test (SAT) and a favourable response to therapy.

## 2. Case Report

**Figure 1:**
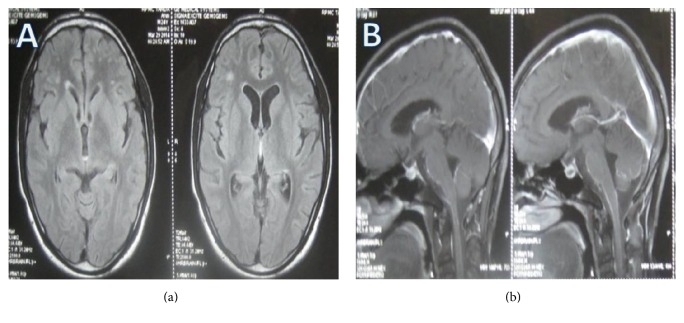
(a) Axial FLAIR images showing white matter periventricular and subcortical hyperintensities bilaterally. (b) After contrast brainstem meningeal enhancement is seen.

**Figure 2:**
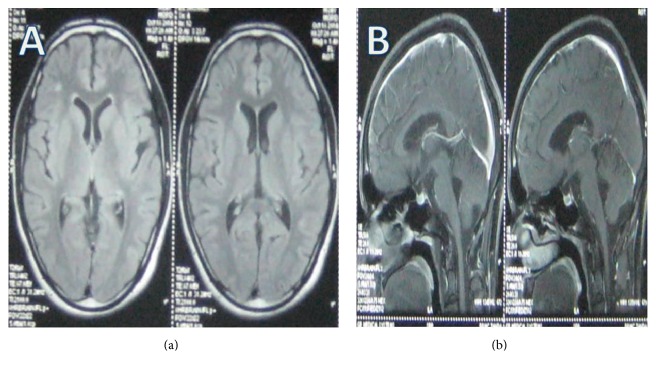
Follow-up MRI 6 months later showing (a) axial FLAIR images showing partially resolved white matter periventricular and subcortical hyperintensities. (b) After contrast, no brainstem meningeal enhancement is seen.

A 24-year-old male was admitted in March 2014, with history of fever for 3 months. The fever was documented up to 103°F and was associated with sweating. There was history of insidious onset, progressive weakness of both lower limbs proximal more than distal in the form of not being able to stand from squatting, climbing up, and getting down stairs for the last 2 months. Simultaneously, he noticed proximal weakness in both upper limbs in the form of inability to lift heavy objects as he used to previously. Patient also gave history of difficulty in walking for the same duration. History of increased frequency of micturition accompanied with urgency and precipitancy was present for the last month. In addition patient had developed impairment in hearing for the last month. No history of headache, vomiting, ear discharge, altered sensorium, or seizures was reported. Review of other systems was normal. No significant past history was present. The patient belonged to a rural area and was associated with livestock rearing. He used to consume raw milk of goat. (This history was disclosed retrospectively.) On examination patient was febrile. Rest of the general physical examination was normal. On nervous system examination, our patient was conscious with the mini-mental state examination score of 28/30. Examination of cranial nerves revealed bilateral sensory neural deafness. Motor examination revealed normal muscle bulk, spasticity in upper and lower limbs, grade IV power in proximal muscles of both upper and lower limbs, symmetrically brisk deep tendon reflexes, and bilaterally extensor plantar response. Sensory examination was normal. Gait was spastic. No meningeal signs were present. Rest of the neurological examination was normal. Review of other systemic examinations was normal. On investigations hemoglobin was 11.6 gm% and total leukocyte count was 7200/cmm. Peripheral smear revealed a microcytic hypochromic picture. Biochemistry showed normal blood glucose and renal and liver functions. Cerebrospinal fluid (CSF) analysis had proteins: 273 mg/dL; glucose: 29 mg/dL (concomitant blood glucose: 101 mg/dL); adenosine deaminase: 19.5 U/L. On microscopic examination of CSF total WBCs count was 288/cmm; neutrophils were 12%; lymphocytes were 88%; total RBCs count was 16/cmm. CSF was VDRL nonreactive, negative for cryptococcal infection and negative for acid fast bacilli by ZN stain. Chest X-ray and ultrasonography of abdomen were normal. As a part of fever workup his blood sample was sent to Department of Veterinary Microbiology, College of Veterinary and Animal Sciences, CSK HPKV Palampur, Himachal Pradesh, for diagnosis of brucellosis. Blood sample was positive for RBT. SAT was positive in 1 : 640 titers. Subsequently brain magnetic resonance imaging (MRI) was done. On T2W and FLAIR images white matter hyperintensities bilaterally involving periventricular white matter and centrum semiovale (mainly involving frontal lobes) with involvement of subcortical U fibres at places were observed. After contrast brainstem meningeal enhancement was seen (Figures 1(a) and 1(b)). On pure tone audiometry bilateral moderate sensorineural hearing loss was documented. Patient was prescribed doxycycline 100 mg twice a day, rifampicin 600 mg once a day, and cotrimoxazole (160 mg trimethoprim and 800 mg sulfamethoxazole) twice a day. Patient became afebrile on fourth day of treatment. All three drugs were continued for three months. At the end of three months patient remained afebrile, deafness recovered, and bladder dysfunction and spastic gait improved. He had resumed his routine work. He faced difficulty only in running around. His upper motor neuron signs persisted on clinical examination. Repeat SAT titer was 1 : 160. So drugs were continued for another three months (total 6 months). Follow-up MRI at 6 months of treatment showed partial resolution of subcortical and periventricular white matter lesions in bilateral inferior frontal regions. Resolution of brainstem meningeal enhancement was also seen (Figures 2(a) and 2(b)). Patient had a sequelae in the form of brisk deep tendon reflexes and bilaterally extensor plantar response after 6 months of therapy which was subsequently stopped. The functional status had a score of 1 on modified Rankin scale.

## 3. Discussion

Brucellosis is considered a deceptive infectious disease in India [7]. Human brucellosis is well reported in India; however there are only few reports on neurobrucellosis [8–10]. Neurological complications of brucellosis are infrequent but an important clinical entity. Clinical presentation of central nervous involvement is variable. Nervous system involvement is generally in meningoencephalitis form. Development of basal meningitis may lead to lymphocytic pleocytosis, cranial nerve involvement, or intracranial hypertension [11]. Guven et al. [4] observed that headache, blurred vision, loss of vision, hearing loss, and confusion were significantly associated with neurobrucellosis. Muscular weakness, disorientation, neck rigidity, changes in deep tendon reflexes, and paresthesias were also more common amid the patients. Among cranial nerves abducens, facial and vestibulocochlear were affected more than other cranial nerves in neurobrucellosis. Peripheral nerve involvement was observed as radiculopathy or polyradiculopathy. Signs and symptoms of meningeal involvement are nonspecific in neurobrucellosis and meningeal signs are infrequently present [11].

*Brucella* bacteria may affect the nervous system directly or indirectly, as a result of cytokine or endotoxin on the neural tissue. Cytotoxic T lymphocytes and microglia activation play an immunopathologic role in this disease. Infection triggers the immune mechanism leading to a demyelinating state of cerebral and spinal cord [11].

In neurobrucellosis imaging findings may be found in four types: normal, meningeal contrast enhancement, white matter changes, and vascular changes [11]. In addition to nonenhancing bilateral white matter lesions deep grey matter involvement has also been documented [10].

Most important differential diagnosis of brucellosis is tuberculosis in our country. Both chronic granulomatous infectious diseases are endemic in our country. There is a clear overlap between neurobrucellosis and tuberculosis both in terms of clinical presentation, laboratory parameters, and neuroimaging. Hearing loss due to vestibulocochlear nerve involvement, deep grey matter involvement, and extensive white matter lesions on neuroimaging mimicking demyelinating disorders seems to be unique for brucellosis [10, 12].

Neurobrucellosis is a diagnostic puzzle as there is a lack of consensus in diagnostic criteria. According to Kochlar et al. [8], the criteria necessary for definite diagnosis of neurobrucellosis are (i) neurological dysfunction not explained by other neurologic diseases, (ii) abnormal CSF indicating lymphocytic pleocytosis and increased protein, (iii) positive CSF culture for* Brucella* organisms or positive* Brucella* IgG agglutination titer in the blood and CSF, and (iv) response to specific chemotherapy with a significant drop in the CSF lymphocyte count and protein concentration. Recently Guven et al. [4] diagnosed neurobrucellosis by the presence of any one of the following criteria: (1) symptoms and signs suspect of neurobrucellosis, (2) isolation of* Brucella* species from cerebrospinal fluid (CSF) and/or presence of anti-*Brucella* antibodies in CSF, (3) the presence of lymphocytosis, increased protein, and decreased glucose levels in the CSF, or (4) findings in cranial MRI or computed tomography (CT). Erdem et al. [13] defined chronic* Brucella* meningitis on the basis of following criteria:

1. The manifestation of clinical neurological symptoms for over 4 weeks
2. The presence of typical CSF evidence with meningitis (protein concentrations >50 mg/dL, pleocytosis over 10/mm^3^, and CSF glucose to serum glucose ratios <0.5)
3. Positive bacterial culture or serological test results for brucellosis in CSF (positive Rose Bengal Test or serum tube agglutination) or in blood (positive Rose Bengal Test and serum tube agglutination with a titer ≥1/160) or positive bone marrow culture
4. Nonappearance of any alternative neurological diagnosis

These criteria were applied in the case definition of 177 patients with chronic brucellar meningitis or meningoencephalitis in a multicenter, retrospective Istanbul 2 study. Based on the results of the study, the sensitivities of the principal serological tests like serum SAT, RBT, and ELISA as well as CSF RBT and SAT were analyzed. The sensitivities of the tests were 94% for serum SAT, 96% for serum RBT, 78% for CSF SAT, and 71% for CSF RBT. The data supported the view that blood serological tests were significantly more sensitive than CSF tests [13]. CSF culture, when positive, is considered the gold standard in the laboratory diagnosis of neurobrucellosis [14]. However, serological approaches are the mainstays in the diagnosis of neurobrucellosis due to the relatively lower efficacy of bacterial culture. Our patient fulfilled all the four criteria of neurobrucellosis as laid in the case definition by Erdem et al. [13].

In patients with consistent clinical features, overemphasis on determination of CSF* Brucella* agglutination titers and isolation of* Brucella* from CSF can be done away in diagnostic criteria with in resource limited settings like our country [12].

There is no consensus for choice of antibiotic, dose, and duration of the treatment for neurobrucellosis. Dual- or triple-combination therapy with doxycycline, rifampicin, trimethoprim-sulfamethoxazole, streptomycin, or ceftriaxone for >2 months (3–6 months) has been recommended [4].

Short-course steroid therapy has been found to be effective in minimizing the residual deficits in those with arachnoiditis, optic neuritis, and multiple-sclerosis-like presentation [15]. Sequelae among survivors despite appropriate antibiotic therapy are well known [4, 9, 16]. They are significant if patient has diffuse CNS, encephalitis, or spinal cord involvement compared to meningitis as a presentation. They have been reported as aphasia, hearing loss, hemiparesis, and visual impairment. Mortality is uncommon [4, 11, 17].

Most of the laboratories lack facilities for diagnosis of human brucellosis in India. In presence of appropriate history and clinical findings, RBT is a very useful test for the diagnosis of human brucellosis. Being simple and affordable it should be an ideal test for diagnosis of brucellosis in patients with clinical setting in our rural hospitals [18].
